# Supplementary material for: NeuroExaminer: an all-glass microfluidic device for whole-brain in vivo imaging in zebrafish
Source: Commun Biol. 2020 Jun 16;3:311. doi: 10.1038/s42003-020-1029-7 (PMC7298014; doi:10.1038/s42003-020-1029-7)
Supplement: Supplementary file 1 — Reporting Summary [file 42003_2020_1029_MOESM1_ESM.pdf]

nature research

Corresponding author(s): Andreas Dietzel

Last updated by author(s): Nov 11, 2019

Reporting Summary

Nature Research wishes to improve the reproducibility of the work that we publish. This form provides structure for consistency and transparency in reporting. For further information on Nature Research policies, see Authors & Referees and the Editorial Policy Checklist.

Statistics

For all statistical analyses, confirm that the following items are present in the figure legend, table legend, main text, or Methods section.

n/a	Confirmed	
☐	☒	The exact sample size (*n*) for each experimental group/condition, given as a discrete number and unit of measurement
☒	☐	A statement on whether measurements were taken from distinct samples or whether the same sample was measured repeatedly
☒	☐	The statistical test(s) used AND whether they are one- or two-sided<br>*Only common tests should be described solely by name; describe more complex techniques in the Methods section.*
☒	☐	A description of all covariates tested
☒	☐	A description of any assumptions or corrections, such as tests of normality and adjustment for multiple comparisons
☒	☐	A full description of the statistical parameters including central tendency (e.g. means) or other basic estimates (e.g. regression coefficient) AND variation (e.g. standard deviation) or associated estimates of uncertainty (e.g. confidence intervals)
☒	☐	For null hypothesis testing, the test statistic (e.g. *F*, *t*, *r*) with confidence intervals, effect sizes, degrees of freedom and *P* value noted<br>*Give P values as exact values whenever suitable.*
☒	☐	For Bayesian analysis, information on the choice of priors and Markov chain Monte Carlo settings
☒	☐	For hierarchical and complex designs, identification of the appropriate level for tests and full reporting of outcomes
☒	☐	Estimates of effect sizes (e.g. Cohen's *d*, Pearson's *r*), indicating how they were calculated

Our web collection on statistics for biologists contains articles on many of the points above.

Software and code

Policy information about availability of computer code

Data collection	Ansys Fluent 18.0 was used for simulation of stimuli application in the microfluidic device (NeuroExaminer); light-sheet data was acquired with LAS X 3.5.2. All the software that was used to collect data is also mentioned in the manuscript.
Data analysis	Data analysis was performed with Imaris 9.1.2 and Fiji 1.5.2c. Prism 7.0e, Quicktime 7.6.3 Pro, Adobe Photoshop CC 19.1.8, and Adobe Illustrator CC 22.1 were used for a graphical representation of the data. All the software that was used to analyze the data is also mentioned in the manuscript.

For manuscripts utilizing custom algorithms or software that are central to the research but not yet described in published literature, software must be made available to editors/reviewers. We strongly encourage code deposition in a community repository (e.g. GitHub). See the Nature Research guidelines for submitting code & software for further information.

Data

Policy information about availability of data

All manuscripts must include a data availability statement. This statement should provide the following information, where applicable:

- Accession codes, unique identifiers, or web links for publicly available datasets
- A list of figures that have associated raw data
- A description of any restrictions on data availability

The authors declare that the data supporting the findings of this study are available within the paper and its supplementary information files.

Field-specific reporting

Please select the one below that is the best fit for your research. If you are not sure, read the appropriate sections before making your selection.

☒ Life sciences ☐ Behavioural & social sciences ☐ Ecological, evolutionary & environmental sciences

For a reference copy of the document with all sections, see nature.com/documents/nr-reporting-summary-flat.pdf

Life sciences study design

All studies must disclose on these points even when the disclosure is negative.

Sample size	Sample size was not predetermined. Imaging quality of zebrafish in the open and closed microfluidic device (NeuroExaminer) was consistent across individual larvae. Thus, a sample size of n ≥ 10 was considered sufficient.
Data exclusions	No data was excluded with the exception of unhealthy zebrafish larvae, e.g. if they displayed an irregular high Ca2+ intensity.
Replication	Imaging of zebrafish larvae in the open and closed microfluidic device (NeuroExaminer) was reproducible across individual fish.
Randomization	Randomization was not relevant to the current study, since there were no different experimental groups. However, individual zebrafish larvae were imaged randomly in either the closed or open microfluidic device (NeuroExaminer).
Blinding	Blinding was neither relevant nor possible in the current study, since no treatments of fish were applied and imaging in the open or closed microfluidic device (NeuroExaminer) was necessarily apparent to the investigator.

Reporting for specific materials, systems and methods

We require information from authors about some types of materials, experimental systems and methods used in many studies. Here, indicate whether each material, system or method listed is relevant to your study. If you are not sure if a list item applies to your research, read the appropriate section before selecting a response.

Materials & experimental systems

n/a	Involved in the study	
☒	☐	Antibodies
☒	☐	Eukaryotic cell lines
☒	☐	Palaeontology
☐	☒	Animals and other organisms
☒	☐	Human research participants
☒	☐	Clinical data

Methods

n/a	Involved in the study	
☒	☐	ChIP-seq
☒	☐	Flow cytometry
☒	☐	MRI-based neuroimaging

Animals and other organisms

Policy information about studies involving animals; ARRIVE guidelines recommended for reporting animal research

Laboratory animals	6 days post fertilization (dpf) Tg(elavl3:H2B-GCaMP6s) zebrafish (Danio rerio) in the crystal background were used in this study.
Wild animals	n/a
Field-collected samples	n/a
Ethics oversight	All animal procedures and experiments were conducted in accordance with the European Union Directive 2010/63/EU to reduce and minimize animal suffering and were approved by the Lower Saxony State Office for Consumer Protection and Food Safety (LAVES) with the animal protocol number 33.19-42502-04-17/2533.

Note that full information on the approval of the study protocol must also be provided in the manuscript.
