# Supplementary material for: NeuroExaminer: an all-glass microfluidic device for whole-brain in vivo imaging in zebrafish
Source: Commun Biol. 2020 Jun 16;3:311. doi: 10.1038/s42003-020-1029-7 (PMC7298014; doi:10.1038/s42003-020-1029-7)
Supplement: Supplementary file 14 — Supplementary Information [file 42003_2020_1029_MOESM14_ESM.pdf]

Supplementary Material

Supplementary Figures

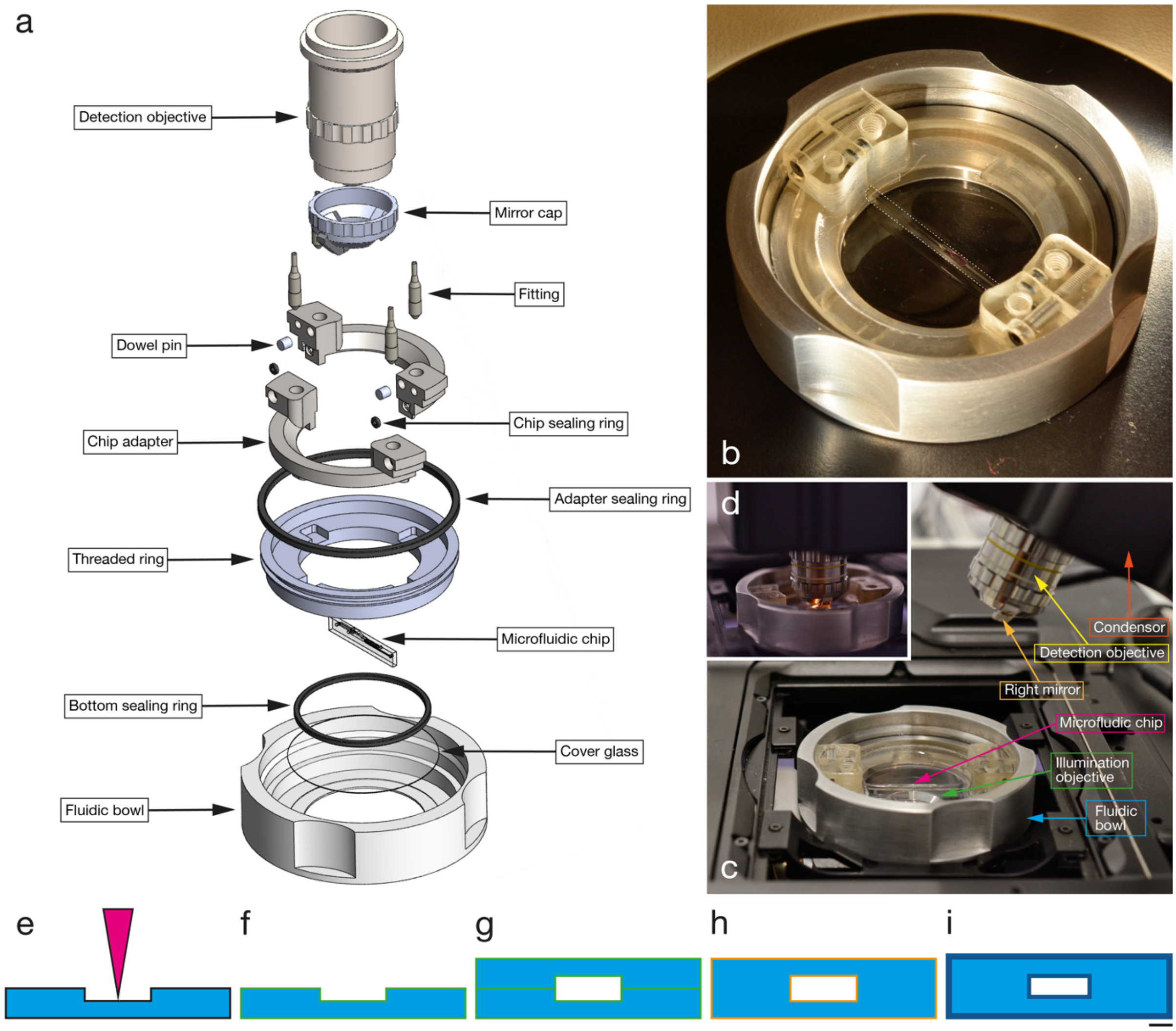

Supplementary Figure 1 (a) Schematic overview of the newly developed components together with the microscope's detection objective and mirror cap. (b) The picture shows the fluidic bowl, the microfluidic chip (NeuroExaminer), and the chip adapter. The microfluidic chip containing the fish sluice is outlined with a dotted white and magenta line, respectively. (c) The microfluidic chip inside the fluidic bowl is depicted on the stage of a Leica SP8 DLS microscope with the condenser on the microscope arm flipped backward. The illumination objective (2.5x, NA 0.07) can be seen below the fluidic bowl and the detection objective (10x, NA 0.3) together with its 5 mm mirror cap that is fixed to the condenser on top. The inset (d) depicts the setup as it looks like during image acquisition, i.e. with the condenser flipped forward so that the two mirrors on the detection objective come to lie on the left and right side of the NeuroExaminer in order to generate a light sheet perpendicular to the top-bottom axis of the microfluidic chip (see also Figure 1g). (e-i) The most important chip fabrication steps are illustrated as: laser ablation (e), chemical etching and cleaning (f), thermal pre-bonding (g), thermal bonding (h) and final thermal treatment for improving optical quality (i). The scale bar in (e-i) is 1 mm.

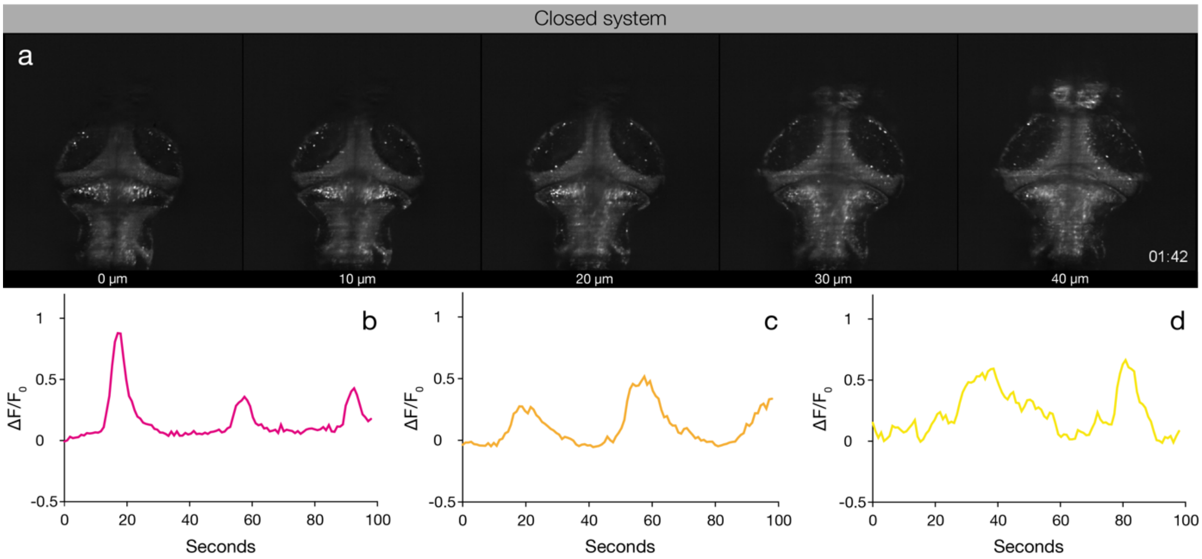

Supplementary Figure 2 (a) 5 optical sections recorded every 10 µm from the brain of a 6 dpf *Tg(elavl3:H2B-GCaMP6s)* larva placed in the closed NeuroExaminer and imaged at 1.1 Hz (see also supplementary movie 9). (b-d) Single-cell color-coded calcium traces (110 frames; ~98 seconds) in the optic tectum (b, magenta), cerebellum (c, orange), and hind brain (d, yellow) extracted from the second (10 µm) plane depicted in (a; see also supplementary movie 10).
