# Supplementary material for: NeuroExaminer: an all-glass microfluidic device for whole-brain in vivo imaging in zebrafish
Source: Commun Biol. 2020 Jun 16;3:311. doi: 10.1038/s42003-020-1029-7 (PMC7298014; doi:10.1038/s42003-020-1029-7)
Supplement: Supplementary file 16 — Description of Additional Supplementary Files [file 42003_2020_1029_MOESM16_ESM.pdf]

Supplementary movie 1 (related to figure 1a, b)

Visualization of transient state simulations (played back at 12 fps) depicting the front of an injected stimulus (red) reaching the larva head in the closed NeuroExaminer version. The time elapsed since entry of the stimulus into the alignment chamber is depicted in lower right corner in seconds. The scale bar is 1 mm.

Supplementary movie 2 (related to figure 1c, d)

Visualization of transient state simulations (played back at 12 fps) depicting the front of an injected stimulus (red) reaching the larva head in the open NeuroExaminer version. The time elapsed since entry of the stimulus into the alignment chamber is depicted in lower right corner in seconds. The scale bar is 1 mm.

Supplementary movie 3 (related to figure 2b)

A time-lapse movie (175 frames; ~10 minutes) of a maximum intensity projection (21 optical sections) of a 6 dpf *Tg(elavl3:H2B-GCaMP6s)*; *crystal* larva imaged in the open system. Time is depicted in lower right corner in min:sec:ms.

Supplementary movie 4 (related to figure 2c, d)

A time-lapse movie (175 frames; ~10 minutes) visualizing all 21 optical planes of a 6 dpf *Tg(elavl3:H2B-GCaMP6s)*; *crystal* larva imaged in the open system. Time is depicted in lower right corner in min:sec:ms.

Supplementary movie 5 (related to figure 2f)

A time-lapse movie (175 frames; ~10 minutes) of a maximum intensity projection (21 optical sections) of a 6 dpf *Tg(elavl3:H2B-GCaMP6s)*; *crystal* larva imaged in the closed system. Time is depicted in lower right corner in min:sec:ms.

Supplementary movie 6 (related to figure 2g, h)

A time-lapse movie (175 frames; ~10 minutes) visualizing all 21 optical planes of a 6 dpf *Tg(elavl3:H2B-GCaMP6s)*; *crystal* larva imaged in the closed system. Time is depicted in lower right corner in min:sec:ms.

Supplementary movie 7 (related to figure 2i-m)

A time-lapse movie (30 frames; ~101 seconds) of a single optical plane (z-plane 8) that was used to generate the calcium traces depicted in figure 2i-m. The arrows in the main frame are color-coded in cyan, green, magenta, orange, and yellow and denote cells in the telencephalon, habenula, optic tectum, cerebellum, and hind brain, respectively. The inset in the upper right corner shows a higher magnification of the analyzed cells. The scale bar is 100 µm and 10 µm in the main frame and insert, respectively. Time is depicted in lower right corner in seconds.

Supplementary movie 8

Two real-time movies (10 seconds; recorded with 30 fps) of a 6 dpf *Tg(elavl3:H2B-GCaMP6s)*; *crystal* larva in the closed microfluidic device before light sheet imaging. The movie on the left outlined in cyan depicts the larva as seen through the oculars of a Leica MZ6 stereo microscope after it has been inserted through the fish sluice; the movie on the right outlined in magenta depicts the same larva as seen through the oculars of a Leica SP8 DLS microscope just before light sheet imaging. The mirrors attached to the detection objective are outlined with a dotted white line. The scale bar is 1 mm. Time is depicted in lower right corner in seconds.

Supplementary movie 9 (related to supplementary figure 2a)

To image with 1.1 Hz (instead of 0.29 Hz for the whole brain) the Leica SP8 DLS is limited to about 40 µm in *z*-depth (5 optical sections each 10 µm). The time-lapse movie (335 frames; ~300 seconds) shows 5 optical planes in the dorsal part of the brain of a 6 dpf *Tg(elavl3:H2B-GCaMP6s)*; *crystal* larva

imaged in the closed system. Note that relatively sharp images can be generated for all of these optical planes. Time is depicted in the lower right corner in min:sec.

Supplementary movie 10 (related to supplementary figure 2b-d)

A time-lapse movie (115 frames; ~103 seconds) of a single optical plane (z-plane 2) that was used to generate the calcium traces depicted in supplementary figure 2b-d. The arrows in the main frame are color-coded in magenta, orange, and yellow and denote cells in the optic tectum, cerebellum, and hind brain, respectively. The inset in the upper right corner shows a higher magnification of the analyzed cells. The scale bar is 100 µm and 10 µm in the main frame and insert, respectively. Time is depicted in lower right corner in seconds.

Supplementary data file legends

Supplementary data file 1

ΔF/F0 values underlying the graphs depicted in figure 2i-m.

Supplementary data file 2

ΔF/F0 values underlying the graphs depicted in supplementary figure 2b-d.
